# The roles of neutrophil serine proteinases in idiopathic inflammatory myopathies

**DOI:** 10.1186/s13075-018-1632-x

**Published:** 2018-07-05

**Authors:** Siming Gao, Xiaoxia Zuo, Di Liu, Yizhi Xiao, Honglin Zhu, Huali Zhang, Hui Luo

**Affiliations:** 1 Department of Rheumatology, Xiangya Hospital, Central South University, 87 Xiangya Road, Changsha, Hunan 410008 People’s Republic of China; 2 Department of Pathophysiology, Xiangya School of Medicine, Central South University, 110 Xiangya Road, Changsha, Hunan 410008 People’s Republic of China

**Keywords:** Dermatomyositis, Polymyositis, Neutrophil serine proteinases, Vascular permeability, Inflammatory cell migration

## Abstract

**Background:**

Dermatomyositis and polymyositis are the best known idiopathic inflammatory myopathies (IIMs). Classic histopathologic findings include the infiltration of inflammatory cells into muscle tissues. Neutrophil serine proteinases (NSPs) are granule-associated enzymes and play roles in inflammatory cell migration by increasing the permeability of vascular endothelial cells. In this study, we aimed to find the roles of NSPs in pathogenesis of IIMs.

**Methods:**

RNA and DNA were isolated to measure the relative expression of NSPs and their methylation levels. The expression of NSPs in serum and muscle tissues was tested by enzyme-linked immunosorbent assay, immunohistochemistry, and immunofluorescence, respectively. Serum from patients was used to culture the human dermal microvascular endothelial cells (HDMECs), and then we observed the influence of serum on expression of VE-cadherin, endothelial cell tube formation, and transendothelial migration of peripheral blood mononuclear cells (PBMCs).

**Results:**

We found that the expression of NSPs was increased in PBMCs, serum, and muscle tissues of IIM patients; these NSPs were hypomethylated in the PBMCs of patients. Serum NSPs were positively correlated with clinical indicators of IIM patients, including lactic dehydrogenase, erythrocyte sedimentation rate, C-reactive protein, immunoglobulin G, immunoglobulin M, and immunoglobulin A. Patients with anti-Jo-1, with anti-Ro-52, or without interstitial lung disease had lower levels of proteinase 3. Serum NSPs degraded the VE-cadherin of HDMECs, and serum NSP application increased the permeability of HDMECs.

**Conclusions:**

Our studies indicate, for the first time, that NSPs play an important role in muscle inflammatory cell infiltration by increasing the permeability of vascular endothelial cells in IIM patients.

**Electronic supplementary material:**

The online version of this article (10.1186/s13075-018-1632-x) contains supplementary material, which is available to authorized users.

## Background

Idiopathic inflammatory myopathies (IIMs) are a group of heterogeneous autoimmune diseases that are accompanied by progressive symmetric muscle weakness, elevated serum levels of muscle enzymes, electromyographic abnormalities, and inflammatory infiltrates observed via muscle biopsy [1, 2]. Dermatomyositis (DM; juvenile, adult), polymyositis (PM), sporadic inclusion body myositis (sIBM), and immune-mediated necrotizing myopathy (IMNM) are the most common subtypes of IIMs [3], of which DM and PM are probably the best known [4].

Classic histopathologic findings for DM are CD4^+^ T cells, B cells, and plasmacytoid dendritic cells (pDCs) infiltrating muscle fibers. Unlike DM, the infiltrating inflammatory cells of PM are CD8^+^ T cells and macrophages [5, 6]. The infiltrating CD4^+^ T cells can differentiate into Th1 and Th17 cells; Th1 can activate macrophages by producing IFN-γ, and Th17 can facilitate the migration of mononuclear cells to the muscle by producing IL-17 [7, 8]. The infiltrating CD8^+^ T cells mediate myocytotoxicity via release of perforin [9]. The infiltrating macrophages can present antigens to T cells and produce multiple cytokines and chemokines [10]. Additionally, infiltrating pDCs may be the main source of type I interferon [11]; however, the exact mechanism by which these inflammatory cells migrate into muscles is not fully understood and requires further study.

Neutrophil serine proteinases (NSPs) are granule-associated enzymes that play an essential role in blood coagulation, apoptosis, inflammation, and immune responses [12–14]. Recent studies have shown that three NSPs—cathepsin G (CTSG), neutrophil elastase (NE), and proteinase 3 (PR3)—play roles in inflammatory cell migration. These NSPs can cleave the endothelial VE-cadherin, are involved in the possible disruption of endothelial integrity [15, 16], and can disturb the endothelial cell cytoskeletal architecture to increase vascular permeability [17, 18]. Moreover, CTSG is a chemoattractant for mononuclear cells and neutrophils [19]. There are few studies about NSPs and their roles in DM/PM.

In our previous microarray analysis, we used total RNA from peripheral blood mononuclear cells (PBMCs) of DM and PM patients, and Illumina HumanHT-12 v4.0 Expression Beadchips (Illumina, Inc.) for mRNA transcription profiling. The platform contained 47,323 transcripts. We identified a total of 2006 differentially expressed genes in patients with DM/PM compared to normal controls (NC) (*P* < 0.05 and fold change ≥ 2), among which 908 genes were upregulated and 1098 genes were downregulated. CTSG, NE, and PR3 were increased in DM and PM patients (data not shown).

In our previous genome-wide DNA methylation analysis, we used the Illumina Human-Methylation 450 K BeadChip array for DNA methylation profiling as described previously [20]. This platform contains 485,000 CpG sites across the whole genome and covers 99% of RefSeq genes with an average of 17 CpG sites per gene region distributed across the promoter, 5′ UTR, first exon, gene body, and 3′ UTR. It covers 96% of CpG islands with additional coverage in island shores and the flanking regions. The results showed that CTSG, NE, and PR3 were hypomethylated in DM and PM patients; the exact methylation sites are presented in Additional file 1: Table S1.

Previous studies have proven that purified NSPs can increase vascular permeability by cleaving the endothelial VE-cadherin. We hypothesize that in DM and PM patients the upregulation of NSPs cleaves the endothelial VE-cadherin, thus disrupting endothelial integrity and increasing the permeability of vascular tissue and the migration of inflammatory cells to extravascular tissue. To better understand the function of these proteinases, we measured their expression in PBMCs, serum, and muscle tissues, and further explored serum NSPs in the pathogenesis of DM and PM. We found that the expression of NSPs was increased in PBMCs, serum, and muscle tissues of DM and PM patients; additionally, the NSPs were hypomethylated in PBMCs in patients. Moreover, serum NSPs from DM/PM patients cleaved the endothelial VE-cadherin, disrupted endothelial integrity, and increased the migration of PBMCs.

## Methods

### Patients and controls

We studied 48 DM patients, 16 PM patients, and 39 normal controls (NC). All patients met the Bohan and Peter diagnostic criteria for DM and PM [21, 22]. This study was approved by the institutional review board at Xiangya Hospital, Central South University of Changsha (Changsha, Hunan, China). All of the participants in the study signed a written informed consent form prior to participation.

### RNA isolation and real-time quantitative PCR

Peripheral blood samples were obtained from patients and controls as described previously [23]. We isolated PBMCs in Ficoll-Paque Plus (GE Healthcare) by density gradient centrifugation. We used Trizol (Invitrogen Life Technologies) to isolate RNA from PBMCs and a Reverse Transcription System (Promega) to obtain cDNA. The relative expression of genes was measured by gene-specific primers (shown in Additional file 2: Table S2) with SYBR Green (SYBR Premix Ex Taq RT-PCR kit; Takara) and the 7500 real-time PCR system analyzer (Applied Biosystems).

### DNA isolation and bisulfite pyrosequencing

Genomic DNA was isolated from PBMCs using genomic DNA extraction kits (Life Technologies, Gaithersburg, MD, USA). We used pyrosequencing to validate the microarray methylation data, and DNA samples were bisulfite-converted by the EpiTect Plus DNA Bisulfite Kit (Qiagen). Bisulfite pyrosequencing was performed on a PyroMark Q96 MD pyrosequencing system with the PyroMark Gold Q96 CDT reagent kit (Qiagen). Gene-specific PCR and sequencing primers were designed by the PyroMark Assay Design 2.0 software (Qiagen). The PCR reaction mixture consisted of 12.5 μl of 2× EPIK Amplification Mix, 0.6 μl (10 μM) primer, 20 ng bisulfite-converted DNA, and added water to a total volume of 25 μl (EPIK Amplification Kit; Bioline). Amplifications were performed with an initial denaturation step at 95 °C for 2 min, 40 cycles of 95 °C for 15 s, 56 °C for 15 s, and 72 °C for 30 s, and finally 4 °C for 20 min. The Pyro Q-CpG software (Qiagen) was used for data analysis.

### Enzyme-linked immunosorbent assay

The expression levels of CTSG, NE, and PR3 in serum were quantified using the human ELISA kit (Abnova) according to the manufacturer’s instructions. The absorbance was measured at 450 nm with a microplate reader.

### Histological analysis and immunohistochemistry

We obtained the muscle biopsy specimens from the bicipital muscles of patients and controls. Muscles were frozen in isopentane cooled with liquid nitrogen. For histological examination, we stained the prepared histological sections (8 μm thick) with hematoxylin and eosin (HE). For immunohistochemistry, frozen sections were fixed by acetone, incubated in 3% hydrogen peroxide to block endogenous peroxidase activity, blocked with 10% goat serum for 30 min, and incubated with rat anti-human CTSG (Abcam) overnight at 4 °C. This was followed by application of the secondary antibody for 40 min, avidin–horseradish peroxidase for 45 min, and DAB chromogen. Finally, the slides were counterstained with hematoxylin as described previously [24].

### Immunofluorescence

After blocking the frozen sections with 5% bovine albumin, the slides were incubated overnight with rat anti-human NE (Abcam) or rabbit anti-human PR3 (Abcam) at 4 °C followed by the secondary antibody for 1 h without light. We then stained the slides with DAPI for 5 min.

### Cell culture and treatment

Human dermal microvascular endothelial cells (HDMECs), which were bought from Cellbio (Catalog #CBR130858), were cultured in Dulbecco’s Modified Eagle’s medium (DMEM; Gibco) supplemented with 10% heat-inactivated fetal bovine serum (Hangzhou Sijiqing Biological Technology) in an atmosphere containing 5% CO_2_ and 95% air at 37 °C as described previously [25]. When cells reached 80–90% confluence, we incubated them in serum-free medium overnight. We then exposed the cells to serum from patients and controls or serum from patients pretreated with phenylmethylsulphonyl fluoride (PMSF) for 30 min. Serum was diluted with culture medium at a 1:5 ratio.

### Western blot analysis

A bicinchoninic acid kit was used to calculate the concentration of total proteins. RIPA buffer was used to lyse HDMECs and the total proteins were then denatured at 100 °C for 10 min. Sodium dodecyl sulfate-polyacrylamide gel electrophoresis (SDS-PAGE) was carried out to isolate the denatured proteins, and the proteins were then transferred to a polyvinylidene difluoride (PVDF) membrane. Five percent skim milk in Tris-buffered saline with Tween (TBST) was used to block the membrane for 1 h, and then the membrane was incubated overnight with rat anti-human VE-cadherin (Abclonal) at 4 °C. The next day, the membrane was washed and incubated with the secondary antibodies. The enhanced chemiluminescence (ECL) method was used to quantify the western blotting results.

### Endothelial cell tube formation

Seventy-five microliters of Matrigel matrix (Corning) were added into each well of 96-well plates and then incubated at 37 °C for 30 min to allow the basement membrane to gel. One hundred microliters of HDMECs (10^6^ cells) were added to each well, and cells were incubated in a 5% CO_2_ humidified incubator for 4 h at 37 °C. The tubes were then observed with an inverted microscope.

### Transendothelial migration of PBMCs

A 24-well Transwell with an 8-μm-pore membrane insert (Corning) was used. PBMCs (10^6^ cells) were added to the top chamber on a confluent monolayer of treated HDMECs. The bottom chamber was filled with 500 μl 1640 Media supplemented with 10% heat-inactivated fetal bovine serum (Hangzhou Sijiqing Biological Technology) and 10 ng/ml MIP-1α (Peprotech). Cells were cultured for 24 h at 37 °C in a 5% CO_2_, humidified incubator. Transmigrated cells were counted in the lower membranes after staining with toluidine blue.

### Statistical analysis

Data are shown as the mean ± SEM. Univariate comparisons were made using a one-way ANOVA or two-sample *t* test. Count data comparisons were made using a chi-squared test. Correlations were made using Pearson’s *r* coefficient. *P* < 0.05 was considered statistically significant. The chi-squared test was performed using SPSS; the other tests were performed using GraphPad Prism software.

## Results

### Clinical and laboratory features of DM/PM patients

All patients and NC were matched for age, sex, and ethnicity (Table 1). DM patients have higher incidence of interstitial lung disease (ILD) than PM patients and PM patients had higher levels of lactic dehydrogenase (LDH) than DM patients, but there was no difference in creatine kinase (CK), erythrocyte sedimentation rate (ESR), C-reactive protein (CRP), immunoglobulin A (IgA), IgM, and IgG, component 3 (C3) and C4, anti-Jo-1 autoantibody, and anti-Ro-52 autoantibody. More DM patients were treated with thalidomide and hydroxychloroquine, which are both effective in the treatment of rashes.

**Table 1:** Clinical manifestations and laboratory data of DM patients, PM patients, and controls

Clinical characteristic	NC (*n* = 39)	DM (*n* = 48)	PM (*n* = 16)	*P* value^a^		
				DM vs NC	PM vs NC	DM vs PM
Age, mean ± SD	48.87 ± 12.40	50.25 ± 11.97	44.41 ± 11.99	0.606	0.2170	0.0889
Sex (male/female)	9/30	11/37	6/10	0.986	0.275	0.253
Disease duration (months)	–	15.41 ± 21.34	23.00 ± 34.25	–	–	0.3138
CK (U/L)^b^	–	842.46 ± 1969.14	4368.46 ± 7149.77	–	–	0.061
LDH (U/L)^c^	–	409.43 ± 267.63	612.02 ± 432.16	–	–	0.032*
ESR (mm/h)^d^	–	57.93 ± 95.67	43.29 ± 34.00	–	–	0.542
CRP (mg/L)^e^	–	14.92 ± 20.72	12.99 ± 23.47	–	–	0.766
IgA (mg/L)^f^	–	2625.22 ± 1408.36	2660.25 ± 1308.82	–	–	0.932
IgM (mg/L)^g^	–	1848.37 ± 1546.84	1715.38 ± 874.10	–	–	0.748
IgG (g/L)^h^	–	29.58 ± 98.34	16.70 ± 7.69	–	–	0.616
C3 (mg/l)^u^	–	888.65 ± 238.43	967.25 ± 319.90	–	–	0.318
C4 (mg/l)^j^	–	243.07 ± 127.62	299.75 ± 347.79	–	–	0.534
Jo-1 (positive/negative)	–	11/37	5/11	–	–	0.505
Ro-52 (positive/negative)	–	16/32	2/14	–	–	0.108
ILD (yes/no)	–	23/23	3/13	–	–	0.029*
Prednisone (yes/no)	–	46/2	16/0	–	–	0.407
Cyclophosphamide (yes/no)	–	6/42	1/15	–	–	0.488
Methotrexate (yes/no)	–	8/40	5/11	–	–	0.209
Azathioprine (yes/no)	–	9/39	5/11	–	–	0.295
Mycophenolate mofetil (yes/no)	–	4/40	0/16	–	–	0.212
Thalidomide (yes/no)	–	25/23	1/15	–	–	0.001*
Hydroxychloroquine (yes/no)	–	29/19	4/12	–	–	0.014*
Total glucosides of *Paeonia* (yes/no)	–	16/32	7/9	–	–	0.452

There were no significant differences in age and gender between NC, DM patients, and PM patients

*NC* normal controls, *DM* dermatomyositis, *PM* polymyositis, *SD* standard deviation

^a^*t* test, *P < 0.05 significant

^b^Normal range of creatine kinase (*CK*): 40–200 U/L

^c^Normal range of lactate dehydrogenase (*LDH*): 120–250 U/L

^d^Normal range of erythrocyte sedimentation rate (*ESR*): 0–20 mm/h

^e^Normal range of C-reactive protein (*CRP*): 0–8 mg/L

^f^Normal range of immunoglobulin A (*IgA*): 690–3820 mg/L

^g^Normal range of immunoglobulin M (IgM): 630–2770 mg/L

^h^Normal range of immunoglobulin G (*IgG*): 7.23–16.85 g/L

^i^Normal range of complement component 3 (*C3*): 850–1930 mg/L

^j^Normal range of complement component 4 (*C4*): 120–360 mg/L

### Expression of NSPs in DM/PM patients and methylation of NSPs

**Fig. 1:**
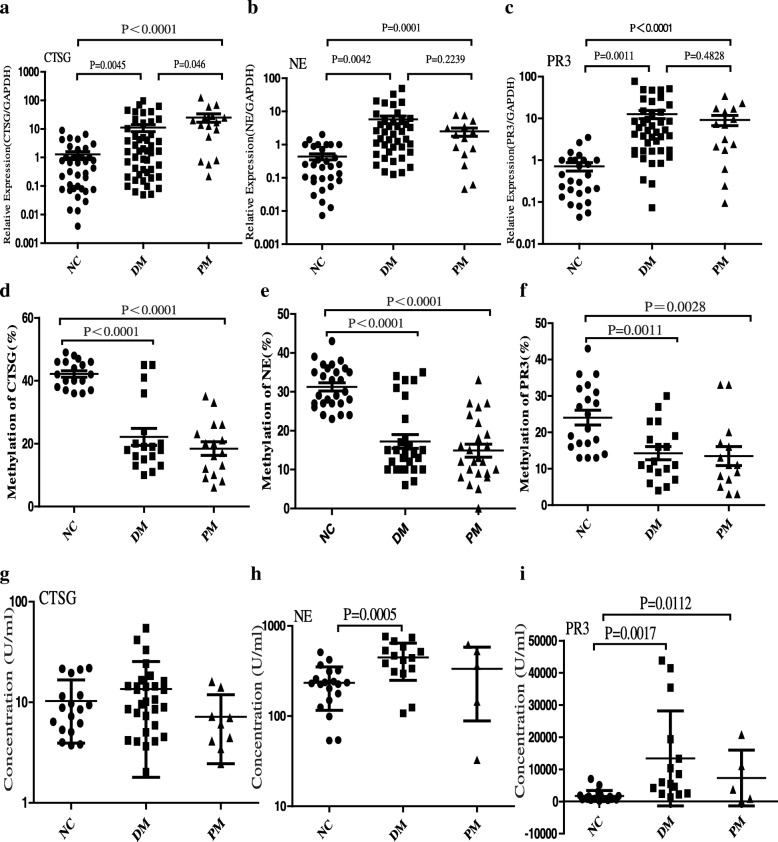
Expression of CTSG, NE, and PR3 in DM/PM and their methylation. Relative expression of CTSG, NE, and PR3 increased in DM and PM PBMCs compared to controls at RNA level (**a**–**c**). CTSG, NE, and PR3 hypomethylated in PBMCs of DM/PM patients (**d**–**f**). No significant difference in serum levels of CTSG in DM, PM, and controls (**g**). Serum levels of NE and PR3 higher in DM patients and PR3 higher in PM patients than in controls (**h**, **i**). Values are mean ± SEM. CTSG cathepsin G, DM dermatomyositis, GAPDH glycerol-3-phosphate dehydrogenase, NC normal control, NE neutrophil elastase, PM polymyositis, PR3 proteinase 3

Real-time PCR was performed to confirm the results of our previous microarray analysis. Our results showed that the relative mRNA levels of CTSG (DM patients 11.09 ± 3.02, PM patients 25.36 ± 8.16), NE (DM patients 5.80 ± 1.54, PM patients 2.50 ± 0.69), and PR3 (DM patients 12.64 ± 2.74, PM patients 9.21 ± 2.54) were significantly upregulated in DM/PM patients compared to those of normal controls (CTSG 1.29 ± 0.32, NE 0.44 ± 0.09, PR3 0.71 ± 0.16) (Fig. 1a–c). The relative expression of CTSG was higher in PM patients than in DM patients (*P* = 0.0046), but there was no difference in the expression of NE (*P* = 0.239) and PR3 (*P* = 0.4828). The results of bisulfite pyrosequencing showed that CTSG (DM patients 22.17 ± 2.70, PM patients 18.44 ± 2.18), NE (DM patients 17.22 ± 1.74, PM patients 14.88 ± 1.67), and PR3 (DM patients 14.28 ± 1.82, PM patients 13.50 ± 2.59) were hypomethylated in DM/PM patients compared to normal controls (CTSG 42.17 ± 1.02, NE 31.26 ± 1.05, PR3 24.05 ± 2.05), but the difference between DM patients and PM patients was not obvious (CTSG *P* = 0.2975, NE *P* = 0.3388, PR3 *P* = 0.8017) (Fig. 1d–f).

CTSG, NE, and PR3 could be released to extracellular space when cells are stimulated. We found that the serum levels of NE (*P* = 0.0005) and PR3 (*P* = 0.0017) were higher in DM patients than in normal controls (Fig. 1h, i), and the levels of PR3 (*P* = 0.0112) were higher in PM patients than in controls. However, the levels of CTSG were almost the same in all three groups (Fig. 1g).

### Expression of NSPs in DM/PM patient muscle tissues

**Fig. 2:**
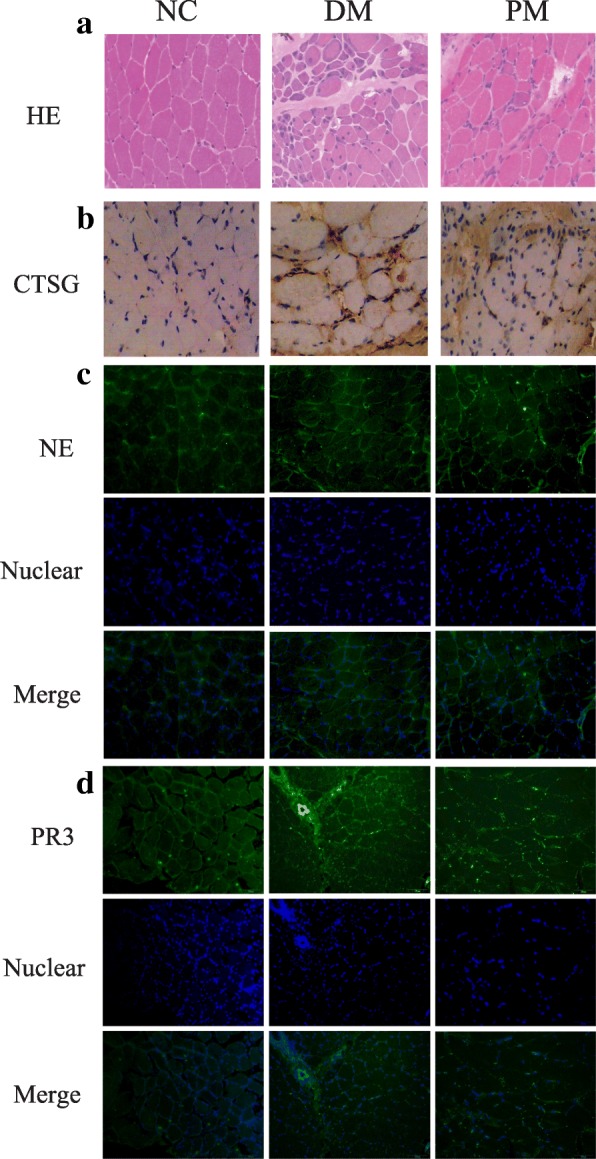
Expression of CTSG, NE, and PR3 in DM/PM muscle tissues. DM sections displayed typical perifascicular atrophy and inflammatory cell infiltration in perimysial area, while inflammatory cells mainly infiltrated around or invaded non-necrotic muscle fibers in HE (**a**). Immunohistochemistry showed an increase of CTSG protein in DM/PM muscle tissues, especially around muscle bundles (**b**). Expression of NE (green) and PR3 (green) also significantly higher in muscle bundles and perivascular areas of DM/PM patients shown by immunofluorescence (**c**, **d**). Nuclei (blue) stained by DAPI. CTSG cathepsin G, DM dermatomyositis, HE hematoxylin and eosin, NC normal control, NE neutrophil elastase, PM polymyositis, PR3 proteinase 3

DM patients displayed typical perifascicular atrophy and inflammatory cell infiltration in the perimysial area. In PM patients, the inflammatory cells mainly existed around or invaded non-necrotic muscle fibers (Fig. 2a). Immunohistochemistry showed that the expression of CTSG was higher in DM/PM patients, especially around myofibers in the muscle tissues. There was no obvious CTSG expression in the controls (Fig. 2b). The expression of NE and PR3 in muscle tissues was also significantly higher in DM/PM patients (Fig. 2c, d), mainly around myofibers and in perivascular areas.

### Correlations between serum NSPs and clinical indicators

**Fig. 3:**
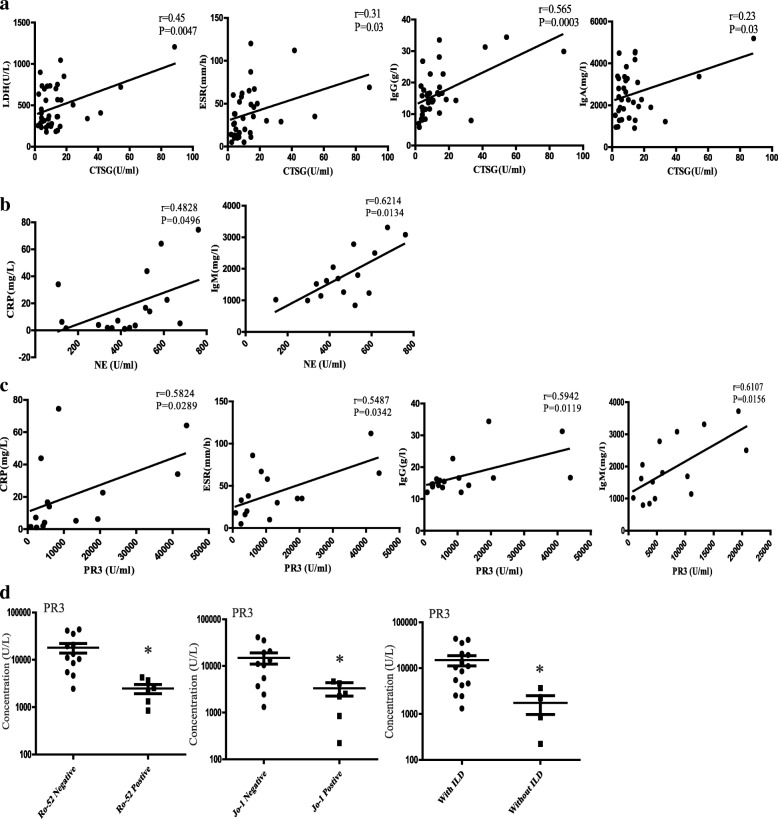
Correlations between serum CTSG, NE, and PR3 levels and clinical indicators. Serum levels of CTSG in DM/PM correlated positively with levels of LDH, ESR, IgG, and IgA (**a**). Serum levels of NE in DM/PM correlated positively with CRP and IgM (**b**). Serum levels of PR3 correlated positively with CRP, ESR, IgG, and IgM in DM/PM patients (**c**). Patients with anti-Jo-1, with anti-Ro-52, or without ILD had lower levels of PR3 (**d**). CRP C-reactive protein, CTSG cathepsin G, ESR erythrocyte sedimentation rate, Ig immunoglobulin, ILD interstitial lung disease, LDH lactic dehydrogenase, NE neutrophil elastase, PR3 proteinase 3. *represents compared with patients, whose anti-Ro-52 antibody or anti-Jo-1 antibody are negative, or patients with ILD *P* < 0.05

To determine the relationship between NSPs and clinical indicators of the diseases, correlation analysis was carried out. The results indicated that the serum levels of CTSG in DM/PM patients were positively correlated with LDH, ESR, IgG, and IgA (Fig. 3a), NE showed positive correlation with CRP and IgM (Fig. 3b), and PR3 had positive correlation with CRP, ESR, IgG, and IgM (Fig. 3c). Patients with anti-Jo-1, with anti-Ro-52, or without ILD had lower levels of PR3 (Fig. 3d). However, the serum levels of CTSG and NE were not significantly different between anti-Jo-1 antibody-positive patients and anti-Jo-1 antibody-negative patients, between anti-Ro-52 antibody-positive patients and anti-Ro-52-negative patients, or between patients with ILD and patients without ILD.

### Serum NSPs degraded VE-cadherin and increased the permeability of HDMECs

**Fig. 4:**
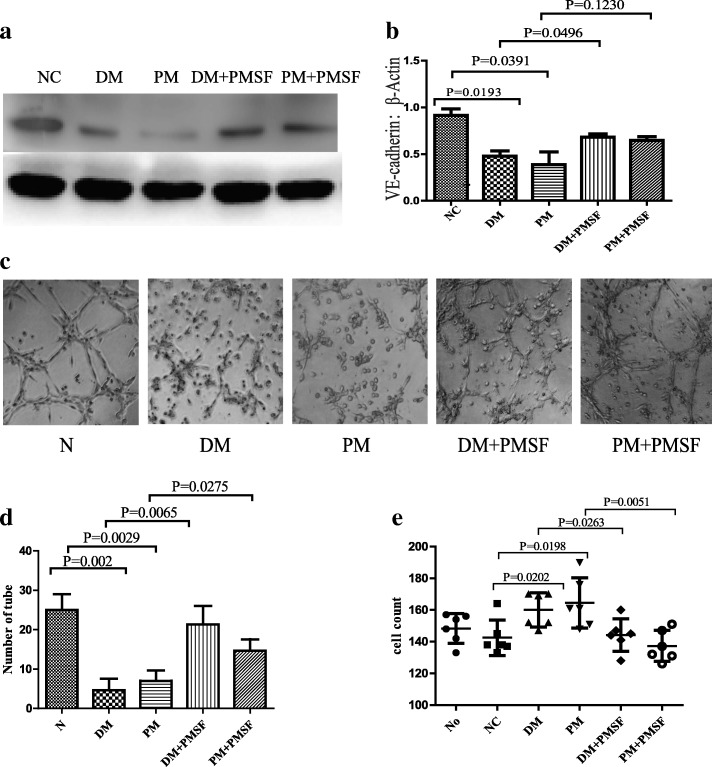
Serum NSPs degraded VE-cadherin, disrupted tube formation, and increased permeability of HDMECs. After stimulating HDMECs with 20% serum from patients for 24 h, expression of VE-cadherin (110 kDa) decreased. VE-Cadherin expression could be neutralized by nonspecific inhibitor PMSF (**a**, **b**). After treating HDMECs with 20% serum from patients, tube formation ability of HDMECs decreased. This function of serum could be alleviated by PMSF (**c**, **d**). After treating HDMECs with 20% serum from patients, permeability of HDMECs to human PBMCs was significantly increased. This increase could be lessened by PMSF (**e**). Values are mean ± SEM. DM dermatomyositis, NC normal control, PM polymyositis, PMSF phenylmethylsulphonyl fluoride

Serum from the patients was applied to HDMEC monolayers to assess its effect on the integrity of junctional proteins. The results showed that serum NSPs significantly degraded VE-cadherin, which could be weakened by their nonspecific inhibitor PMSF (Fig. 4a, b). Serum NSPs could disrupt the tube formation of HDMECs (Fig. 4c, d). Serum NSPs increased the permeability of HDMECs to human PBMCs, yet this increase was attenuated by PMSF (Fig. 4e).

## Discussion

We reported that the relative expression of NSPs was significantly higher and hypomethylated in the PBMCs of DM/PM patients. NE and PR3 were elevated in DM/PM serum, and there was positive correlation between serum NSPs and clinical indicators, such as ILD, LDH, ESR, CRP, IgG, IgM, IgA, anti-Jo-1 autoantibody, and anti-Ro-52 autoantibody. Serum NSPs degraded the VE-cadherin of HDMECs while increasing the permeability of HDMECs. This study is the first to measure the expression of CTSG, NE, and PR3 in DM/PM patients and to show human dermal microvascular endothelial junctional protein degradation and increased permeability.

In our study, DM patients have the higher incidence of ILD. The serum level of LDH was significantly higher in PM patients than in DM patients, and the level of CK had an increasing trend. These findings were in accordance with previous studies because PM patients typically have more severe muscle disease [26, 27]. DM can be distinguished from PM by its typical cutaneous features, which include heliotrope rash, Gottron’s papules, V-sign rash, shawl sign rash, and so on [28, 29]. Thalidomide has biologic effects on cytokines and cell-mediated responses, and is beneficial in systemic lupus erythematosus, pyoderma gangrenosum, erythema nodosum leprosum, and skin manifestation of DM [30, 31]. Hydroxychloroquine also has a positive effect on the cutaneous manifestations of DM [32]. Our DM patients had higher utilization rates of thalidomide and hydroxychloroquine for the treatment of skin lesions related to DM.

CTSG, NE, and PR3 are three serine proteinases that are stored in azurophil granules of neutrophils, monocytes, mast cells, and so on [33]. When the aforementioned cells were stimulated by immune complexes, certain pharmacological agents, or phagocytosis, the three NSPs were either released to the extracellular space or bound to the surface of those cells [33, 34]. These three NSPs exert several effects, such as processing inflammatory mediators and extracellular matrix proteins, thrombus formation, and engaging protease-activated receptors [14]. Our study proves for the first time that NSPs were increased in PBMCs, serum, and muscle tissues of DM/PM patients; that the serum levels of NSPs had a positive correlation with the levels of LDH, ESR, CRP, IgG, IgA, and IgM; and that patients with ILD have higher levels of PR3. It has been reported that the serum LDH level has significant correlation with the degree of muscle weakness, muscle destruction, and the degree of muscle inflammation [35], and that the levels of LDH tend to indicate disease activity [27]. Serum levels of ESR and CRP are risk factors of interstitial lung disease (ILD) in DM/PM [36–39], elevated ESR is associated with increased mortality in patients with DM [37, 40], and CRP has positive correlation with the global activity scores in DM [41]. So, to an extent, these parameters can reflect the activity or prognosis of DM/PM. Further study is needed to identify the exact functions of NSPs in DM/PM patients.

Recently, studies have shown that NSPs can enter the endothelial cell, disturb the endothelial cytoskeletal architecture, increase endothelial cell apoptosis, and reduce viability [17, 42]. NE can disrupt junctional proteins such as E-cadherin and VE-cadherin [43] and β-catenin [44], and can break down components of the extracellular matrix [45]. NE also activates matrix metalloproteinase 9 [46], thus potentiating the destructive effects on the vasculature and facilitating neutrophil transmigration [47]. PR3 has been shown to induce the release of CXCL-8 from endothelial cells [48], to activate matrix metalloproteinases [49] and to potentiate neutrophil transmigration across the endothelium [50]. Serum NSPs degraded VE-cadherin in the HDMECs in vitro. VE-cadherin is a critical component of the adherens junction [51]. Homogeneous VE-cadherin interactions with adjacent cells maintain cell–cell adhesion and contribute to endothelial permeability. Loss of VE-cadherin function greatly reduces microvascular stability and leads to increased permeability and hemorrhage [52, 53]. Degradation of protein junctions would result in increased permeability of endothelial cells.

What is the clinical relevance of our findings? The infiltration of inflammatory cells into muscles plays a key role in the pathogenesis of DM/PM. We identified NSPs as key molecules that degrade primary junctional proteins of the HDMECs, thereby increasing permeability of the HDMECs and facilitating the migration of inflammatory cells. In light of these findings, selective NSP antagonists, with or without antioxidants, may offer future vascular protection and new treatment for DM/PM.

Despite the novel and clinically relevant findings in this study, there are some limitations. First, the patients included in our study differed in the course and severity of their disease, and they had different treatment options. Second, we used the serum of patients to stimulate HDMECs, which would not completely rule out the influence of other elements in the serum and the NSPs produced by other cells. Third, the function of NSPs was not confirmed in vivo. Thus, further studies should be done to confirm whether the expression and roles of NSPs in patients have relationships with the course and/or severity of the diseases, and the function of NSPs in the model of DM/PM.

## Conclusions

Our study showed that NSPs were elevated in DM/PM patients both at RNA and protein levels. The serum NSPs had some relation with clinical indicators, although the exact relationship was not fully understood. Most importantly, serum NSPs degraded the VE-cadherin of HDMECs and increased the permeability of HDMECs to PBMCs. These interactions may play key roles in the development and progression of DM/PM.

## Additional files

Additional file 1: **Table S1.** Exact methylation sites of CTSG, NE, and PR3 in genome-wide DNA methylation analysis. (DOCX 14 kb)

Additional file 2: **Table S2.** Sequences of specific primers of CTSG, NE, PR3, and GAPDH gene used in our study. (DOCX 14 kb)

## Abbreviations

C3: Component 3
C4: Component 4
CK: Creatine kinase
CRP: C-reactive protein
CTSG: Cathepsin G
DM: Dermatomyositis
DMEM: Dulbecco’s modified Eagle’s medium
ECL: Enhanced chemiluminescence
ELISA: Enzyme-linked immunosorbent assay
ESR: Erythrocyte sedimentation rate
HDMEC: Human dermal microvascular endothelial cell
HE: Hematoxylin and eosin
IgA: Immunoglobulin A
IgG: Immunoglobulin G
IgM: Immunoglobulin M
IIM: Idiopathic inflammatory myopathy
ILD: Interstitial lung disease
IMNM: Immune-mediated necrotizing myopathy
LDH: Lactic dehydrogenase
NC: Normal controls
NE: Neutrophil elastase
NSP: Neutrophil serine proteinase
PBMC: Peripheral blood mononuclear cell
pDC: Plasmacytoid dendritic cell
PM: Polymyositis
PMSF: Phenylmethylsulphonyl fluoride
PR3: Proteinase 3
PVDF: Polyvinylidene difluoride
SDS-PAGE: Sodium dodecyl sulfate-polyacrylamide gel electrophoresis
sIBM: Sporadic inclusion body myositis
TBST: Tris-buffered saline with Tween
