# Supplementary material for: The roles of neutrophil serine proteinases in idiopathic inflammatory myopathies
Source: Arthritis Res Ther. 2018 Jul 5;20:134. doi: 10.1186/s13075-018-1632-x (PMC6034343; doi:10.1186/s13075-018-1632-x)
Supplement: Supplementary file 1 — Table S1. Exact methylation sites of CTSG, NE, and PR3 in genome-wide DNA methylation analysis. (DOCX 14 kb) [file 13075_2018_1632_MOESM1_ESM.docx]

Table S1 Methylation sites of CTSG, NE, and PR3 in genome-wide DNA methylation analysis

| Gene | Methylation sites |
| --- | --- |
| CTSG  NE  PR3 | cg01099512_TSS200, cg01697902_TSS1500, cg01932691_TSS200, cg03502982_Body, cg10082165_Body, cg11189107_3'UTR, cg14932313_Body, cg24355048_Body, cg24777950_TSS1500  cg01963696_TSS1500, cg02487452_Body, cg03526702_Body, cg03633458_TSS200, cg04382396_5'UTR(1^st^ Exon), cg06100973_TSS200, cg06406619_TSS1500, cg07239938_Body, cg08223924_Body, cg08269974_Body, cg11683663_Body, cg14052235_Body, cg15530560_TSS1500, cg19628469_Body, cg21586152_Body, cg22761077_TSS1500, cg26717554_Body  cg00615241_TSS1500, cg02082462_3'UTR, cg03004350_Body, cg05257528_Body, cg09134726_Body, cg09930046_TSS1500, cg10599438_Body, cg12230709_TSS200, cg15760529_Body, cg17004025_TSS200, cg17483523_TSS1500, cg19463169_Body, cg19787694_Body, cg20094462_3'UTR, cg21462596_Body, cg23357789_3'UTR, cg23386659_TSS200, cg23525853_Body, cg24304425_5'UTR(1^st^ Exon), cg24347562_TSS1500, cg27234823_Body, cg27535410_Body |
