# Supplementary material for: The roles of neutrophil serine proteinases in idiopathic inflammatory myopathies
Source: Arthritis Res Ther. 2018 Jul 5;20:134. doi: 10.1186/s13075-018-1632-x (PMC6034343; doi:10.1186/s13075-018-1632-x)
Supplement: Supplementary file 2 — Table S2. Sequences of specific primers of CTSG, NE, PR3, and GAPDH gene used in our study. (DOCX 14 kb) [file 13075_2018_1632_MOESM2_ESM.docx]

Table S2 Sequences of specific primers used in this study

| Gene | Species | Forward primer (5’ to 3’) | Reverse primer (5’ to 3’) |
| --- | --- | --- | --- |
| GAPDH  CTSG  NE  PR3 | Human  Human  Human  Human | GCACCGTCAAGGCTGAGAAC  ACACCCAGCAACACATCACTGC  TGCGCCCAACTTCGTCATGTCG  CATTTGCACTTTCGTCCCTCGC | TGGTGAAGACGCCAGTGGA  GGTTCACGTTTCGATTCCGTCTG  CGTAGCCGTTTTCGAAGATGCG  CATTTGCACTTTCGTCCCTCGC |
